# Comparing synaptic proteomes across five mouse models for autism reveals converging molecular similarities including deficits in oxidative phosphorylation and Rho GTPase signaling

**DOI:** 10.3389/fnagi.2023.1152562

**Published:** 2023-05-15

**Authors:** Abigail U. Carbonell, Carmen Freire-Cobo, Ilana V. Deyneko, Saunil Dobariya, Hediye Erdjument-Bromage, Amy E. Clipperton-Allen, Damon T. Page, Thomas A. Neubert, Bryen A. Jordan

**Affiliations:** ^1^Dominick P. Purpura Department of Neuroscience, Albert Einstein College of Medicine, Bronx, NY, United States; ^2^Department of Neuroscience and Physiology, New York University Grossman School of Medicine, New York, NY, United States; ^3^Department of Neuroscience, The Scripps Research Institute Florida, Jupiter, FL, United States; ^4^Department of Psychiatry and Behavioral Sciences, Albert Einstein College of Medicine, Bronx, NY, United States

**Keywords:** proteomic convergence, PSD, Rho GTPase, Rac, tandem mass tags, TMT, comparative analyses

## Abstract

Specific and effective treatments for autism spectrum disorder (ASD) are lacking due to a poor understanding of disease mechanisms. Here we test the idea that similarities between diverse ASD mouse models are caused by deficits in common molecular pathways at neuronal synapses. To do this, we leverage the availability of multiple genetic models of ASD that exhibit shared synaptic and behavioral deficits and use quantitative mass spectrometry with isobaric tandem mass tagging (TMT) to compare their hippocampal synaptic proteomes. Comparative analyses of mouse models for Fragile X syndrome (*Fmr1* knockout), cortical dysplasia focal epilepsy syndrome (*Cntnap2* knockout), *PTEN* hamartoma tumor syndrome (*Pten* haploinsufficiency), ANKS1B syndrome (*Anks1b* haploinsufficiency), and idiopathic autism (BTBR+) revealed several common altered cellular and molecular pathways at the synapse, including changes in oxidative phosphorylation, and Rho family small GTPase signaling. Functional validation of one of these aberrant pathways, Rac1 signaling, confirms that the *ANKS1B* model displays altered Rac1 activity counter to that observed in other models, as predicted by the bioinformatic analyses. Overall similarity analyses reveal clusters of synaptic profiles, which may form the basis for molecular subtypes that explain genetic heterogeneity in ASD despite a common clinical diagnosis. Our results suggest that ASD-linked susceptibility genes ultimately converge on common signaling pathways regulating synaptic function and propose that these points of convergence are key to understanding the pathogenesis of this disorder.

## Introduction

Autism spectrum disorder (ASD) is a neurodevelopmental disorder characterized by social-communication deficits and restrictive and repetitive behaviors. Although the specific cause of ASD is unknown, autism is highly heritable, with a monozygotic twin concordance rate of 40–80% (Gaugler et al., 2014). The genetic architecture of ASD is extraordinarily complex, with common inherited variants and rare *de novo* mutations working together to confer genetic risk (Weiner et al., 2017). This polygenic etiology presents challenges for elucidating the molecular pathogenesis of autism. However, ASD-related syndromes with defined genetic causes for autistic phenotypes present the best opportunities for elucidating the underlying mechanisms of ASD and identifying possible therapeutic targets (Sztainberg and Zoghbi, 2016). These monogenic syndromes, including Fragile X syndrome (*FMR1*), Rett syndrome (*MECP2*), *PTEN* hamartoma tumor syndrome (*PTEN*), tuberous sclerosis complex (*TSC1*/*TSC2*), Phelan McDermid syndrome (*SHANK3*), and cortical dysplasia focal epilepsy syndrome (*CNTNAP2*), play a key role in furthering our understanding of ASD.

Animal models of these syndromes have proven essential for studying the underlying neuropathology of ASD, especially changes in the complex processes of mammalian brain development and function. Studies in rodents have shown that ASD risk genes converge on transcriptional regulation, protein homeostasis, and synaptic structure and function (De Rubeis et al., 2014; Pinto et al., 2014; Ruzzo et al., 2019). Accordingly, mice demonstrating the loss of *Fmr1*, *Mecp2*, *Pten*, *Tsc1/Tsc2*, *Shank* genes, or the *Nrxn* and *Nlgn* families all demonstrate changes in synaptic excitability or plasticity, and most also show altered dendritic growth or spine dynamics (Hulbert and Jiang, 2016; Parikshak et al., 2016; Varghese et al., 2017; Verma et al., 2019). Non-syndromic ASD models, such as those induced by valproic acid or maternal immune activation, also reveal structural and functional synaptic deficits, showing that environmental factors can lead to similar synaptic phenotypes (Sui and Chen, 2012; Martin and Manzoni, 2014; Cellot et al., 2016; Patrich et al., 2016; Li et al., 2018; Wang et al., 2018; Andoh et al., 2019).

We recently characterized *ANKS1B* haploinsufficiency, a new genetic syndrome presenting with autism and other neurodevelopmental disorders that is caused by a monogenic deletion in the *ANKS1B* gene (Carbonell et al., 2019). This gene, which encodes for the protein AIDA-1, was previously identified in ASD risk gene networks (Li et al., 2014). To facilitate our work, we developed a mouse model for *ANKS1B* haploinsufficiency and found that it recapitulates behavioral correlates of the syndrome (Carbonell et al., 2019). Altogether, we found that AIDA-1 regulates activity-induced protein synthesis (Jordan et al., 2007), hippocampal synaptic plasticity (Tindi et al., 2015), and NMDA receptor subunit composition (Tindi et al., 2015; Carbonell et al., 2019). AIDA-1 is a core protein of the postsynaptic density and interacts with PSD95 in a complex that contains other factors associated with neurodevelopmental disorders, including *Grin2b*, *Syngap1*, and *Nlgn* (Kaizuka and Takumi, 2018; Carbonell et al., 2019). Our research provides an additional example corroborating the idea that molecular mechanisms regulating synaptic function underlie ASD pathobiology (Zoghbi and Bear, 2012; Bourgeron, 2015).

Despite the heterogeneous genetic architecture of autism and a complex etiology with contributions from environmental factors, ASD is diagnosed by distinct clinical criteria. Therefore, convergent cellular processes at the circuit, synaptic, or molecular level could underlie these shared behavioral phenotypes (Sestan and State, 2018). In seeking convergent mechanisms among syndromic and non-syndromic forms of autism, comparative studies often narrowly focus on selected behaviors, specific synaptic phenomenology, or shared responses to preclinical pharmacological interventions (Barnes et al., 2015; Heise et al., 2018; Schoen et al., 2019). While broader comparisons have been made using transcriptomic analyses (Forés-Martos et al., 2019; Quesnel-Vallières et al., 2019; Hernandez et al., 2020), these changes may not reflect mechanisms of disease due to multiple downstream levels of regulation such as protein translation, degradation, and transport (Vogel and Marcotte, 2012). Indeed, widespread discrepancies between transcript and protein abundance are well-known (Wang et al., 2017; Hoogendijk et al., 2019) but typically ignored when drawing conclusions from discovery-based transcriptomic screens. Proteomic approaches can therefore yield dramatically different results from transcriptomic profiles, as shown in a mouse model of Rett syndrome (Pacheco et al., 2017), and are particularly favored for ASD-linked genes that regulate protein translation (*Fmr1*, *Tsc1*/*Tsc2*, *Pten*) or degradation (*Ube3a*; Louros and Osterweil, 2016). ASD models in which synaptic scaffolding and membrane localization are altered can also be investigated at the proteomic level, especially when the synaptic compartments and complexes are isolated by fractionation or immunoprecipitation (Lee et al., 2017; Reim et al., 2017; Brown et al., 2018; Murtaza et al., 2020). Indeed, analysis of postmortem brain tissue from ASD patients showed that some transcriptomic changes were not observed while changes in synaptic proteins predicted by ASD risk genes and animal models were confirmed (Abraham et al., 2019).

Here, we compare the postsynaptic proteomes of five mouse models for autism using a particularly rigorous quantitative proteomic method employing isobaric tags. The models selected display synaptic deficits and represent models of ASD-related syndromes Fragile X syndrome (*Fmr1* -/Y), *PTEN* hamartoma tumor syndrome (*Pten* +/−), cortical dysplasia focal epilepsy syndrome (*Cntnap2* −/−), ANKS1B syndrome (*Anks1b* +/−), and the BTBR+ inbred model of idiopathic autism. These models have demonstrated face validity for autism, displaying hallmark behavioral correlates of ASD including social interaction deficits and restrictive behaviors (Kazdoba et al., 2016; Zhou et al., 2016; Kabitzke et al., 2018; Carbonell et al., 2019). In each model, we find evidence for upstream regulators that alter synaptic composition with predicted functional effects consistent with clinical phenotypes in ASD and individual syndromes. Mouse models can be clustered according to similarities and differences in upstream regulators, functional effects, and canonical pathways predicted by changes in their synaptic proteomes. Notably, we identified groups of models with shared changes in the synaptic proteome, which may indicate the presence molecular subtypes of ASD. Among diverse and shared molecular deficits observed in all ASD models tested, deficits in oxidative phosphorylation, and RhoA family signaling were predicted by bioinformatic analyses, and functional assays for Rac1 activity corroborate model-specific bioinformatic predictions. An appreciation of convergent synaptic changes underlying ASD is critical for defining pathogenic mechanisms and prioritizing treatments that can have broad efficacy (Sestan and State, 2018). Our results identify specific druggable pathways that may lead to the design of effective therapeutic interventions for diverse forms of ASD.

## Materials and methods

### Animal models and fractionation

*Fmr1* (stock #003025, MGI:1857169) and *Cntnap2* knockout (stock #017482, MGI: 2677631) mouse models and the BTBR+ (stock #002282), B6129SF2/J (stock #101045), and C57BL/6J (stock #000664) mouse strains were purchased from the Jackson Laboratory. Mice with *Pten* haploinsufficiency (MGI:2151804) and wild-type mice on the C57BL/6J background were obtained from the Page lab at the Scripps Research Institute Florida. Heterozygotes from the *Anks1b* conditional knockout line (stock #035048, MGI:5779292; Tindi et al., 2015) and wild-type mice from the *Nestin-cre* transgenic line (stock #003771, MGI:2176173) were bred in house (Carbonell et al., 2019). Mice from this line expressing the *Nestin-cre* transgene were genotyped for the *Anks1b^wt^* allele (forward: 5′-CACCCACAGCTCCATAGACAG-3′, reverse: 5′-GCACCTATTCCCTTCACCCTG-3′) and *Anks1b^fl^* allele (forward: 5′-AGTTGCCAGCCATCTGTTGT-3′, reverse: 5′-GGGTTCCGGATCAGCTTGAT-3′). Postsynaptic density (PSD) enriched fractions were isolated based on the method of Carlin et al. (1980) and Cohen et al. (1977) as we have done before (Jordan et al., 2004, 2007; Zhang et al., 2012; Tindi et al., 2015). Briefly, mice were euthanized in compliance with the Institutional Animal Care and Use Committee of the Albert Einstein College of medicine and both hippocampi were rapidly removed, placed in ice-cold solution A (0.32 M sucrose, 10 mM HEPES pH 7.4, 1 mM MgCl_2_, 0.5 mM CaCl_2_, protease and phosphatase inhibitors, and 0.1 mM PMSF), and Dounce homogenized. Samples were then centrifuged at 800*g* for 10 min to remove nuclei and other cellular debris, and the supernatant containing light membrane fractions was subjected to a second centrifugation at 30,000*g* for 25 min. The pellet was then resuspended in solution B (0.32 M sucrose, 10 mM HEPES pH 7.4) and layered on top of a 0.85, 1, and 1.2 M sucrose step gradient and centrifuged at 82,500*g* for 2 h. The synaptosomal fraction was collected at the 1–1.2 M sucrose interface and lysed using an equal volume of 1% Triton X-100 (in 0.32 M sucrose, 12 mM Tris pH 8.1) at 4°C for 15 min. Lysed synaptosomes were then centrifuged at 120,000*g* for 25 min to collect the PSD-enriched fraction.

### Antibodies

SDS-PAGE and Western blot were performed under standard conditions using the LI-COR fluorescence-based system with the following antibodies: rabbit anti-Rac1 1:1,000 (Proteintech #24702-1-AP), rabbit anti-RhoA 1:1,000 (ABclonal), rabbit anti-Cdc42 (ABclonal), mouse anti-PSD95 1:1,000 (NeuroMab), and rat anti-tubulin 1:1,000 (Cell Signaling Tech). Statistical analysis for Western blot was performed in JMP 16 (SAS).

### Tandem mass tag labeling of peptides and determination of labeling efficiency

PSD-enriched samples isolated from each mouse model were electrophoresed briefly (dye front about 5 mm beyond the gel well) using a 4–12% SDS-PAGE gel to remove SDS and other mass spectrometry (MS)-incompatible chemicals. Proteins were visualized by staining overnight with GelCode^®^ Coomassie blue reagent (Pierce). A gel fragment containing all stacked protein bands was excised, reduced with DTT, alkylated with iodoacetamide, and digested using 5 ng/μL mass spectrometry-grade trypsin (Trypsin Gold, Promega). The resulting peptides were desalted using a Stage Tip manually packed with Empora C18 High Performance Extraction Disks (3 M) (Rappsilber et al., 2007) and eluted peptide solutions were dried under vacuum. Peptides were then resuspended in 18 μL acetonitrile (ACN), 57 μL of 0.2 M HEPES pH 8.5 was added to each sample and were reacted with unique isobaric labels within a 10-plex tandem mass tag set (TMT10-plex; Thompson et al., 2003). TMT10-plex amine reactive reagents (Thermo Fisher, 5 mg per vial) were re-suspended in 1024 μL anhydrous acetonitrile and 25 μL of reagent was added to each sample (TMT label: peptide [w/w] = 12:1). The mixture was incubated at RT for 1 h, quenched by the addition of 10 μL 5% hydroxylamine for 15 min, and acidified by the addition of 10 μL 10% formic acid. To calculate labeling efficiency, a 5-μL aliquot from each reaction was desalted on a StageTip, analyzed by LC–MS/MS with a Q-Exactive Orbitrap HF (high field), and the resulting spectra searched with MaxQuant using its corresponding TMT label as variable modifications on the N-terminus and lysine residues. Labeling efficiency was 95% or greater for all samples labeled (Supplementary Figure 5). To ensure that equal amounts of labeled peptides from each channel were mixed together, a two-step mixing strategy was employed: in the first step, an identical ~1 μL volume of peptides from each channel was mixed and analyzed, and the value of the median ratio (median of the ratios of all peptide intensities of one channel over their corresponding peptide average intensities of all channels) for each channel was determined as the correction factor. In the second step, the rest of the peptides were mixed by adjusting their volume using the correction factors. In this way, median ratios ranging from 0.97 to 1.02 were achieved as previously reported (Erdjument-Bromage et al., 2018). The final mixture of reaction products from 10 TMT channels were desalted on a Sep-Pak tC18 1 mL Vac Cartridge (Waters, #WAT03820). Eluted peptides were dried by vacuum centrifugation and stored at −20°C.

### Hydrophilic interaction liquid chromatography fractionation of peptides

The TMT-labeled peptides from the PSD samples were then fractionated by offline HILIC to increase depth of coverage of synaptic proteins and to decrease ratio compression resulting from co-fragmenting peptides (Huang et al., 2017). To do this, the final TMT mixture was dissolved in 90% acetonitrile with 0.1% TFA and peptide separation was carried out on an Agilent pump equipped with a TSK gel amide-80 column (4.6 mm ID, 25 cm long) from TOSOH Bioscience, LLC, PA, United States. A gradient of 90% acetonitrile with 0.1% TFA was introduced over 65 min, and a fraction was collected every 2 min. Concatenated pools of peptides (10 pools) were finally created by pooling non-adjacent peptide fractions; about 10% of each pool was used for LC–MS/MS analysis.

### Liquid chromatography–tandem mass spectrometry

Online chromatography was performed with a Thermo Easy nLC 1000 ultrahigh-pressure UPLC system (Thermo Fisher) coupled online to a Q-Exactive HF with a NanoFlex source (Thermo Fisher). Analytical columns (~23 cm long and 75 μm inner diameter) were packed in-house with ReproSil-Pur C18 AQ 3 μm reversed-phase resin (Dr. Maisch GmbH, Ammerbuch-Entringen). The analytical column was placed in a column heater (Sonation GmbH, Biberach) regulated to a temperature of 45°C. The TMT peptide mixture was loaded onto the analytical column with buffer A (0.1% formic acid) at a maximum backpressure of 300 bar. Peptides were eluted with a 2-step gradient of 3–40% buffer B (100% ACN and 0.1% formic acid) in 180 min and 40–90% B in 20 min, at a flow rate of 250 nL/min over 200 min using a 1D online LC–MS2 data-dependent analysis (DDA) method as follows: MS data were acquired using a data-dependent top-10 method, dynamically choosing the most abundant not-yet-sequenced precursor ions from the survey scans (300–1,750 Th). Peptide fragmentation was performed via higher energy collisional dissociation with a target value of 1 × 10^5^ ions determined with predictive automatic gain control. Isolation of precursors was performed with a window of 1 Th. Survey scans were acquired at a resolution of 120,000 at *m*/*z* 200. Resolution for HCD spectra was set to 60,000 at *m*/*z* 200 with a maximum ion injection time of 128 ms. The normalized collision energy was 35. The underfill ratio specifying the minimum percentage of the target ion value likely to be reached at the maximum fill time was defined as 0.1%. Precursor ions with single, unassigned, or seven and higher charge states were excluded from fragmentation selection. Dynamic exclusion time was set at 30 s. Each of the TMT 10-plex samples was analyzed in triplicate.

All data were analyzed with the MaxQuant proteomics data analysis workflow (version 1.5.5.7) with the Andromeda search engine (Cox et al., 2011; Tyanova et al., 2016). The type of the group specific analysis was set to Reporter ion MS2 with 10plex TMT as isobaric labels for Q Exactive HF MS2 data. Reporter ion mass tolerance was set to 0.01 Da, with activated Precursor Intensity Fraction (PIF) value set at 0.75. False discovery rate (the rate at which identified positives are null) was set to 1% for protein, peptide spectrum match, and site decoy fraction levels. Peptides were required to have a minimum length of eight amino acids and a maximum mass of 4,600 Da. MaxQuant was used to score fragmentation scans for identification based on a search with an allowed mass deviation of the precursor ion of up to 4.5 ppm after time-dependent mass calibration. The allowed fragment mass deviation was 20 ppm. MS2 spectra were used by Andromeda within MaxQuant to search the UniProt mouse database (01092015, 16,699 entries) combined with 262 common contaminants. Enzyme specificity was set as C-terminal to arginine and lysine, and a maximum of two missed cleavages were allowed. Carbamidomethylation of cysteine was set as a fixed modification and N-terminal protein acetylation, deamidated (N, Q), and oxidation (M) as variable modifications. The reporter ion intensities were defined as intensities multiplied by injection time (to obtain the total signal) for each isobaric labeling channel summed over all MS/MS spectra matching to the protein group as previously validated (Tyanova et al., 2016). Following MaxQuant analysis, the protein and peptide .txt files were imported into Perseus (version 1.5.6.0) software which was used for statistical analysis of all the proteins identified.

### Experimental samples and bioinformatic analyses

ASD models (*Fmr1*, *Pten*, *Cntnap2*, *Anks1b*, BTBR+) and appropriate controls (C57BL/6J, B6129SF2/J X *Nestin-cre* C57BL/6J) for each model were analyzed in biological triplicates, representing 24 independent samples. Each biological replicate was comprised enriched hippocampal PSD fractions pooled from 2 male mice of each genotype at 6–10 weeks of age (*N* = 3 biological replicates using a total of 6 mice for each genotype). As we could only label 10 samples at a time using the 10-plex TMT isobaric tags, the samples (*Fmr1*, *Pten*, *Cntnap2*, *Anks1b*, BTBR+ models, C57BL/6J controls, and *Nestin-cre* C57BL/6J controls; in triplicate) were analyzed in three independent LC–MS/MS runs, with each run containing the animal model and corresponding control.

Following the identification of proteins and TMT-dependent quantitation of their abundance in each sample, the fold-change was calculated by dividing the relative abundance of a protein in the ASD sample by its abundance in the appropriate control sample. Fold-changes were calculated only between samples within each independent MS analyses. For the *Fmr1*, *Pten*, *Cntnap2*, and BTBR+ models, we used the average of 2 samples of C57BL/6J mice as the control values. For the *Anks1b* Het mice on the *Nestin-cre* C57BL/6J background, we used wild-type littermates on the *Nestin-cre* C57BL/6J background as the control value. The fold-changes were plotted, and values were normalized to a value of 1. To increase the rigor of subsequent analyses, we filtered the protein sets in each experiment for proteins identified and quantified by at least 3 unique peptides, and with a Q-value of 0, representing the highest significance for intensity and score in MaxQuant. We used this list of protein as input in StringDB under the species *Mus musculus* for gene ontology (GO) enrichments analyses (Szklarczyk et al., 2015).

Functional annotation, activation prediction, and regulatory network construction were performed for each mouse model using Ingenuity Pathway Analysis (IPA, QIAGEN Bioinformatics, Fall Release 2022). Fold-change values for each protein in the PSD proteome were used as input for a *Core Analysis* in IPA using the following default settings: Expression Analysis; General Settings = Ingenuity Knowledge Base (Genes Only), Direct Relationships; Networks = Interaction networks, include endogenous chemicals; Node Types = All; Data Sources = All; Confidence = Experimentally Observed; Species = All; Tissues & Cell Lines = All; Mutations = All. To score similarity between models overall and in each domain (Upstream Regulators, Downstream Effects, and Canonical Pathways), *Analysis Match* was used. In *Analysis Match*, activity signatures for each *Core Analysis* are generated by taking the top 50 (Upstream Regulators and Downstream Effects) or 10 (Canonical Pathways) entities that are activated (*z*-score > 2) and inhibited (*z*-score < −2) in each domain. Similarity *z*-scores between Core Analyses are defined using the following formula, where *N* is the total number of overlapping entities in each analysis, *N*_+_ is the number of correct matches, and *N*_−_ the number of incorrect matches:


$$\mathrm{raw}\;z\mathrm{-score}=\left(N_{+}-N_{-}\right)/\left(\mathrm{Sqrt}\left(N\right)\right)$$

The raw *z*-score is divided by a hypothetical perfect match (*N*_+_ = *N* <= 100, *N*_−_ = 0) and multiplied by 100% to obtain a normalized *z*-score, where a score of 100 is a perfect match to itself. Raw and normalized *z*-scores are negative when *N*_−_ > *N*_+_, yielding an opposite activation signature. To generate overall *value of p* scores, the −log_10_ of the *p*-values (maximum value of 50) were calculated for each domain and expressed as a percentage of the maximum possible −log_10_*p* (a perfect match to itself). To compare models, the *Core Analysis* of each model were used as input for a *Comparison Analysis* in IPA. Hierarchical analysis was used to cluster annotations in Upstream Analysis, Downstream Effects, and Canonical Pathways, and to cluster models for similarity relationships.

### G-LISA

G-protein linked immunoassays (G-LISA) were used to quantify active Rac1 in hippocampal tissue lysates. Active Rac1 substrate (the G-protein binding domain of Pak1) fused to GST protein (GST-Pak1) was generated in bacteria and purified using glutathione beads. This substrate was added at a concentration of 1 μg to 50 mM Ammonium Carbonate and absorbed onto high-binding 96-well plates overnight to create binding surfaces for GTP-bound Rac1. Hippocampal tissues from the five different mouse models were prepared using the method described by the Cytoskeleton Inc. BK-128 Kit. (*N* = 6 each of BTBR+, *Fmr1*, and *Cntnap2* mice, *N* = 5 *Pten* mice, *N* = 9 *Anks1b* mice, and *N* = 9 each of the two control Mice, C57BL/6J and B6129SF2/J X *Nestin-cre* C57BL/6J mice were used, all prepared from 6 to 10 weeks of age). Briefly, tissues were lysed for 1 min in the kit lysis buffer, and lysates were snap-frozen to preserve GTP-bound active Rac1. All steps were conducted on ice. Lysates were then incubated on the GST-Pak1 coated 96-wells for exactly 30 min on ice. Primary and secondary antibodies as well as HRP-substrate were provided by the BK-128 Kit, and active Rac1 was detected by standard ELISA using immunodetection at 490 OD and HRP-substrate colorimetric measurements.

## Results

### TMT-MS analyses of postsynaptic density fractions from mouse models of autism

**Figure 1:**
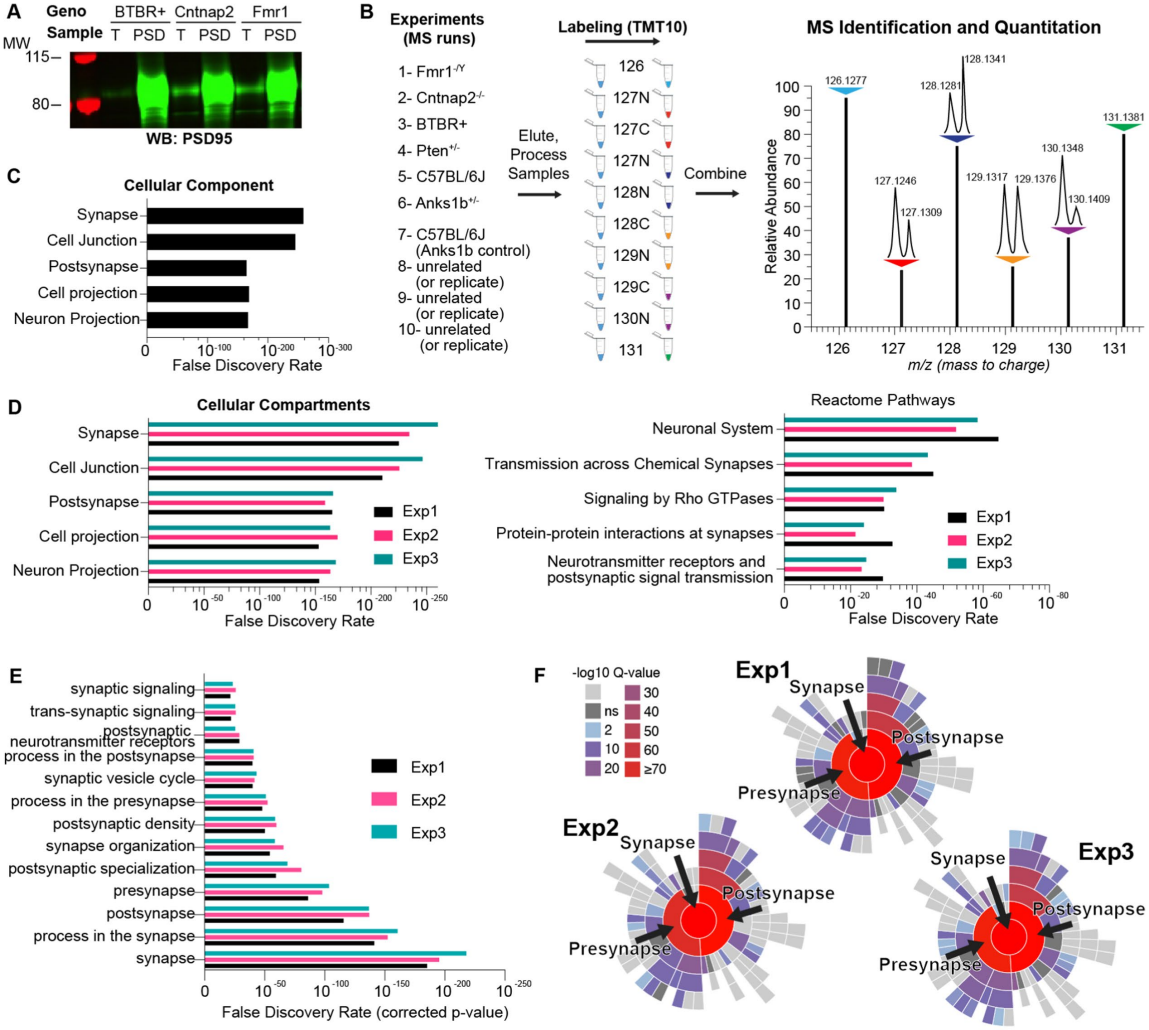
Methodology for isobaric-tag based quantitative comparison of synaptic fractions of ASD mouse models. **(A)** Synapse-enriched fractions from sample mouse models of autism spectrum disorder (ASD) demonstrate qualitative enrichment for the synaptic marker PSD95 by Western blot (4 μg each sample) compared to total brain fractions (T). **(B)** Isobaric labeling and comparative proteomics schematic showing the ASD mouse models used (De Rubeis et al., 2014; Gaugler et al., 2014; Sztainberg and Zoghbi, 2016; Weiner et al., 2017; Ruzzo et al., 2019) and appropriate control samples (Pinto et al., 2014; Hulbert and Jiang, 2016; Parikshak et al., 2016; Varghese et al., 2017; Verma et al., 2019) modified from the schematic from the Thermo Scientific Catalog for the TMT10plex™ Isobaric Kit. Samples were reacted with unique isobaric tags, whose identifier (e.g., 128 N) corresponds to the molecular weight (Daltons) and stable isotope amino acid (N or C) present in that tag. (Right) Idealized MS output showing the same protein in each of the 10 samples identified as 10 contiguous peaks. This permits immediate and rigorous relative quantitation across all samples. m/z represents mass/charge ratio of peptides. *N* = 6 total animals for each genotype. **(C)** Bar graph representing the top cellular components sorted by statistical significance (False Discovery Rate), identified by gene ontology (GO) analysis of proteins in a sample MS run (Exp 1) and using all genes as a search space. **(D)** Same as figure **C** but for showing top Cellular Compartment and Reactome Pathways for each MS run. **(E)** Enrichment analyses using SynGO and using only brain-specific genes as a search space. **(F)** Hierarchical plots showing the top GO terms enriched in each experiment and associated *q*-values (*p*-values adjusted for multiple comparisons) for each term.

To compare the synaptic proteomes from different ASD mouse models, we obtained *Fmr1* knockout (*Fmr1* KO; Consortium, T.D.-B.F.X, 1994), *Pten* haploinsufficiency (*Pten* Het) (Clipperton-Allen and Page, 2014; Clipperton-Allen and Page, 2015), *Cntnap2* knockout (*Cntnap2* KO; Peñagarikano et al., 2011), *Anks1b* haploinsufficiency (*Anks1b* Het; Carbonell et al., 2019), and the BTBR+ inbred mouse strains (McFarlane et al., 2008). All selected models display abnormal behaviors in domains relevant to ASD, including social approach and interaction, stereotyped movements, learning and memory, and sensorimotor function. These models have also been used to illustrate synaptic dysfunction, including altered synapse formation, excitatory/inhibitory balance, synaptic plasticity, and glutamatergic signaling (Hulbert and Jiang, 2016; Varghese et al., 2017; Bagni and Zukin, 2019; Carbonell et al., 2019). We analyzed changes in the synaptic proteomes of hippocampal postsynaptic density (PSD)-enriched fractions isolated from each of the five mouse models of ASD. PSD-enriched fractions were collected as we have done before (Jordan et al., 2004, 2007; Zhang et al., 2012; Tindi et al., 2015; see Methods) and modified to a single Triton X-100 extraction step (Tindi et al., 2015) as opposed to two. This allowed us to collect enough material from mouse hippocampi at the expense of purity. However, Western blots show that isolated fractions were highly enriched for the post synaptic marker PSD95 (Figure 1A). Moreover, the proteins identified in the three independent experiments (described below) show ~50–80% overlap with the Genes to Cognition database of PSD components (G2Cdb; Croning et al., 2009) and ~30–50% overlap with the Synaptic Gene Ontologies curated synaptic database (SynGO) (Koopmans et al., 2019; Supplementary Table 1). Proteins in each sample were then labeled with a unique isobaric tag within a 10-plex Tandem Mass Tag set (TMT). TMTs are reactive isobaric tags with unique permutations of 14C and 15N stable isotopes that imbue peptides with small differences in mass that can then be resolved by mass spectrometry (MS) (Figure 1B). This approach allows for up to 10 samples to be mixed and analyzed simultaneously by MS, which reduces variability, enables robust quantitation of identified proteins, and allows for rigorous quantitative and comparative analyses between samples. Overall, this method allowed us to overcome poor quantitation traditionally associated with MS-based proteomics. The proteins identified in each run, along with number of unique peptides, scores, and other MS-based information are listed in Supplementary Table 1. Using all genes as a search space, gene ontology (GO) analyses using STRING (Szklarczyk et al., 2019) revealed that isolated fractions are overwhelmingly enriched for synaptic terms (Figure 1C). GO analyses also revealed a similar distribution of Cellular Compartments and Reactome Pathways (Figure 1D) across each of the three independent MS experiments, and that each of the specific pathways was enriched to a similar extent. We used SynGO (Koopmans et al., 2019) to restrict the search space to brain-specific genes and found that *Synapse*, *Process in the synapse*, and *Postsynapse* were the most significant GO terms (Figure 1E). Hierarchical plots for GO terms show an overwhelming enrichment (high Q-scores) for synaptic processes for all three experiments (Figure 1F). Overall, these results show that all experimental samples were highly enriched in synaptic components and were similar to each other, which increases the strength of subsequent comparisons.

### Broad comparison of synaptic proteomes across ASD mouse models shows evidence of molecular subtypes

**Figure 2:**
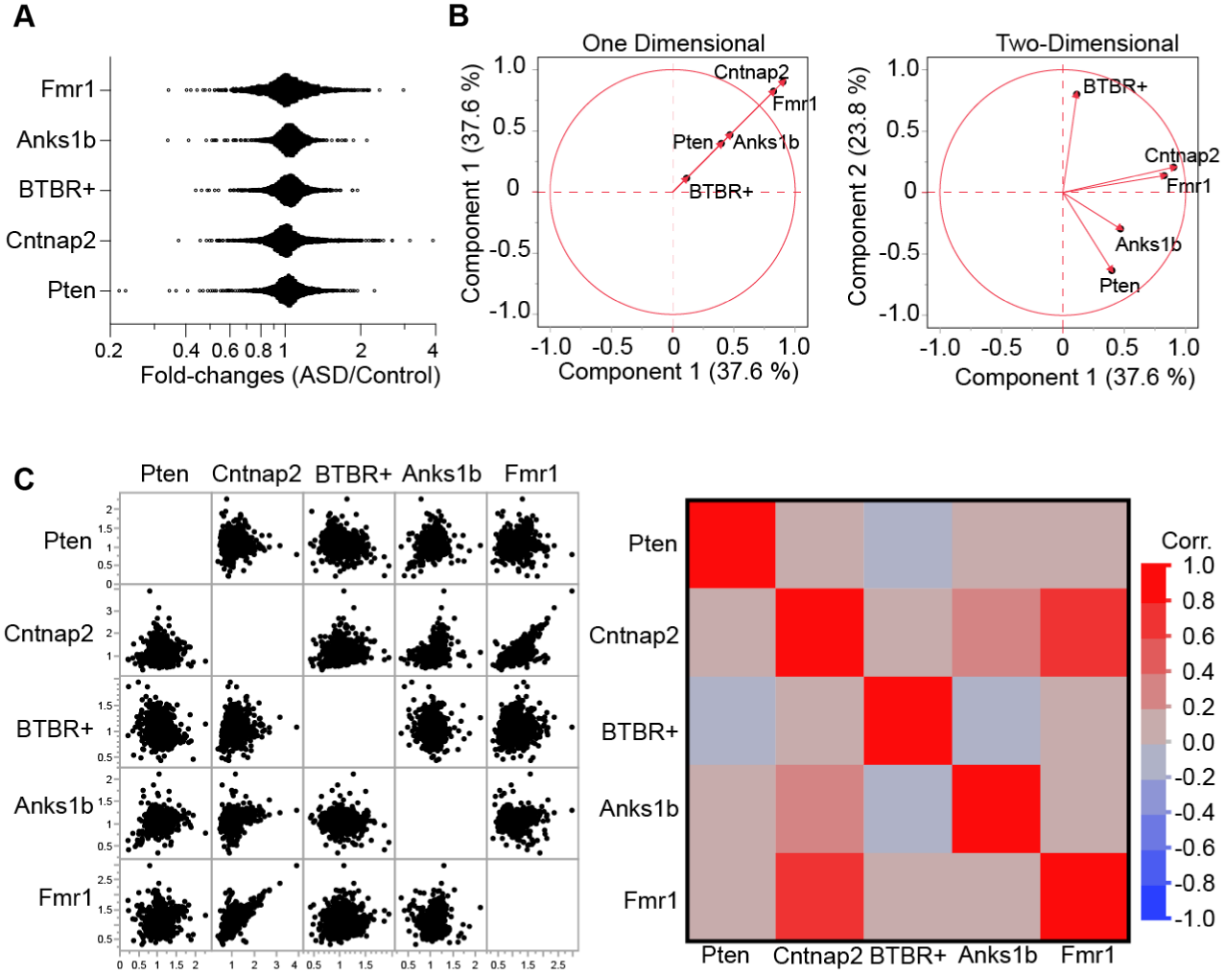
Similarity analyses between mouse models of ASD. **(A)** Distribution histograms for all proteins identified each animal model. Results show that fold-values show distribution with outliers. **(B)** Principal components analysis for the five ASD models plotted across (left) one and (right) two dimensions reveal broad similarities between *Cntnap2* and *Fmr1* models, as well as between *Anks1b* and *Pten* models. **(C)** Correlation matrices (left- data points, right- Pearson’s correlation) based on genes and fold-changes. For all panels, proteins identified = ~1,300–1,500 from *n* = 3 MS runs of *n* = 6 mice per genotype.

To compare findings across ASD models, we first characterized each model by calculating the ratio of synaptic protein abundance in the model to its appropriate control (fold-change). C57BL/6J (duplicates) mice were used as controls for all models except for the *Anks1b* mice, which used wildtype B6129SF2/J X *Nestin-cre* C57BL/6J controls from the same colony. To preserve the quantitative rigor afforded by the isobaric tags, fold-changes were only calculated within experiments (between samples in each independent MS analysis). Moreover, we used a highly stringent filter to increase the significance of findings, considering only proteins identified and quantified by at least 3 unique peptides, and whose MaxQuant calculated Q-values were 0, representing the lowest possible false discovery rate. Finally, only proteins that were identified and quantified in at least 2 independent experiments were considered for subsequent analyses (Supplementary Table 2; 97.6 and 91.2% of proteins found and quantified in Experiment 1 were found and quantified in Experiments 2 and 3, respectively). Distribution plots of protein fold-changes revealed that most proteins were unchanged at hippocampal synapses in the ASD mouse models (fold-changes were normally distributed across a value of 1; Figure 2A). This is expected as large changes in the synaptic proteome would likely be lethal. To measure the overall similarities across models, we performed multivariate analyses using PCA (Principal Components Analyses; Figure 2B) and calculated correlations using protein identities and fold-changes (Figure 2C). Results show that *Fmr1* and *Cntnap2* models are most similar, with *Anks1b* and *Pten* models appearing to comprise a second molecular subgroup. The inbred BTBR+ strain was most dissimilar.

### Dysregulated molecular pathways for oxidative metabolism and Rho GTPase signaling are shared across ASD mouse models

**Figure 3:**
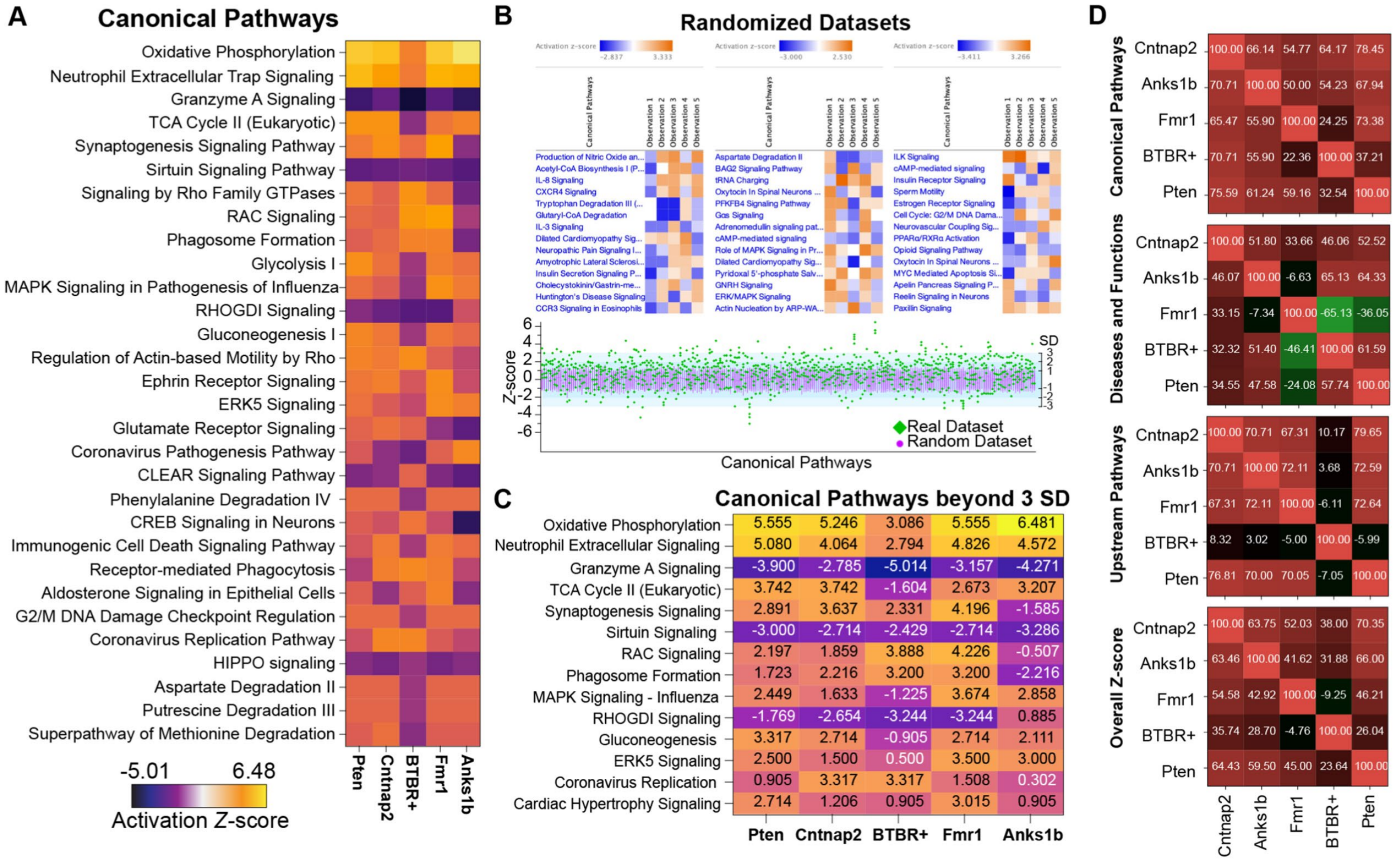
Distinct pattern of similarities and differences in Canonical Pathways among mouse models of autism. **(A)** Heat maps showing the predicted activation or inhibition (see LUT legend) of the top 30 significant (*z*-scores) canonical pathways shared in all ASD models. **(B)** Twenty datasets with randomized and normally distributed fold-ratio values were analyzed by IPA. Results reveal top enriched pathways as expected, but with significantly lower *z*-scores, and no pathways show changes in the same direction (*z*-score) for all ASD models. (lower) Plots of *z*-scores (*Y*-axis) for real datasets (green diamonds) for the identified canonical pathways (*X*-axis). Overlaid are the distribution of *z*-scores for the randomized datasets (pink circles; mean ± SD). A significant number of outliers were identified in the real datasets (green diamonds outside the light blue area). **(C)** Heat map showing the outlier canonical pathways (>3 standard deviations away from the mean of randomized dataset in at least 2 animal models). **(D)** Analysis Match in IPA to determine the similarities between models based on shared canonical pathways, upstream and downstream regulators. Numbers represent correlation values with 100% representing perfect overlap and −100% being anti-correlated. Results track the broad similarities seen in Figure 2.

To analyze the functional implications of the altered synaptic proteomes observed, we used Ingenuity Pathway Analysis (IPA, QIAGEN Bioinformatics). IPA derives functional insights and pathway nomenclatures from a continuously updated knowledge base drawn from the literature (Krämer et al., 2014). We first performed a *Comparison Analyses* in IPA. Sorted by overall *z*-scores (standard deviation away from expected values), we found that several canonical pathways were altered across all models in a highly significant manner (Figure 3A; Supplementary Table 3). Moreover, several pathways were predicted to be regulated in the same way (either activated or inhibited) across all ASD models. We hypothesize that these canonical pathways represent critical or potentially causative molecular mechanisms in autism that could serve as novel targets for therapeutic intervention. All tested models show strongly activated (positive *z*-scores) oxidative phosphorylation and strongly downregulated Granzyme A, Sirtuin, RhoGDI, and HIPPO signaling pathways. These results corroborate long-standing research linking neurodevelopmental disorders to mitochondrial dysfunction (oxidative phosphorylation; Giulivi et al., 2010; Rossignol and Frye, 2012) immune regulatory systems (Granzyme A, Sirtuin; Careaga et al., 2017; Meltzer and Van de Water, 2017; Hughes et al., 2018), and cell proliferation and apoptosis (Granzyme A, HIPPO, RhoGDI, Sirtuin; Dong et al., 2018; Courchesne et al., 2019). In addition, our analyses identified specific dysregulated molecules that may represent the core mechanisms linked to deficits within these broad cellular pathways (Supplementary Table 4). Sirtuins, like granzyme signaling, have been implicated in neuroprotection, cognition, and metabolism, as well as Alzheimer’s disease and aging (Bonda et al., 2011). Other pathways that were altered in all five models showed multi-directional dysregulation. As expected, based on prevailing literature and samples tested, synaptogenesis signaling and glutamate receptor pathways were also significantly affected in all ASD models, corroborating recent hypotheses classifying ASDs as synaptopathies. We also found a significant number of canonical pathways linked to the activity of the small G-protein family RhoA. Interestingly, these pathways showed a similar pattern where the *Anks1b* model was downregulated while the other four models were upregulated.

While IPA incorporates *p*-values, *z*-scores, and other analyses to address statistical biases based on population subsets (Krämer et al., 2014), the exact methodology is proprietary. To independently test the significance of our findings, we created 20 randomized datasets containing the same proteins as in our real samples, but with randomly assigned fold-ratios. These datasets were generated to reflect the distribution and standard deviations calculated from the real datasets of each animal model. IPA analyses of randomized datasets revealed “significant” canonical pathways, but with *z*-score ranges that were substantially lower than those calculated for the actual sample datasets (average random *z*-score range = ± ~ 2.5; average real *z*-score range = −5.01 to 6.48; Figure 3B). Plotting the *z*-scores for the actual dataset together with the mean and standard deviation of the 20 randomized datasets reveals outlier canonical pathways that were at least three standard deviations removed from the mean (Figure 3B). The standard deviations and *z*-scores calculated for both actual and randomized data were almost identical, suggesting a standardized method for calculating expectation values. Pathways that were greater than three standard deviations removed from the mean include Oxidative phosphorylation, Sirtuin signaling, Granzyme A signaling, Synaptogenesis, and Rho signaling pathways, among others (Figure 3C).

We then used *Analysis Match* to create a similarity matrix for all ASD models tested based on Upstream Regulators, Downstream Effects (Diseases and Functions), Canonical Pathways from each *Core Analysis,* and overall *z*-scores (Figure 3D; Supplementary Figures 1-3). The top pathways in each category and for each animal model were based on *p*-values, *z*-scores, and activation values (Krämer et al., 2014; Supplementary Table 3). Similarity *z*-scores reflect the percentage of maximum similarity to a given model (out of 100%, a perfect match to itself). *Analysis Match* based on functional annotations reveals significant similarities across ASD models, which corroborates results presented in Figure 2 and shows that BTBR+ mice represent a distinct molecular subgroup.

### Altered expression and function of Rho GTPases in ASD mouse models validates bioinformatic analyses of synaptic proteomes

**Figure 4:**
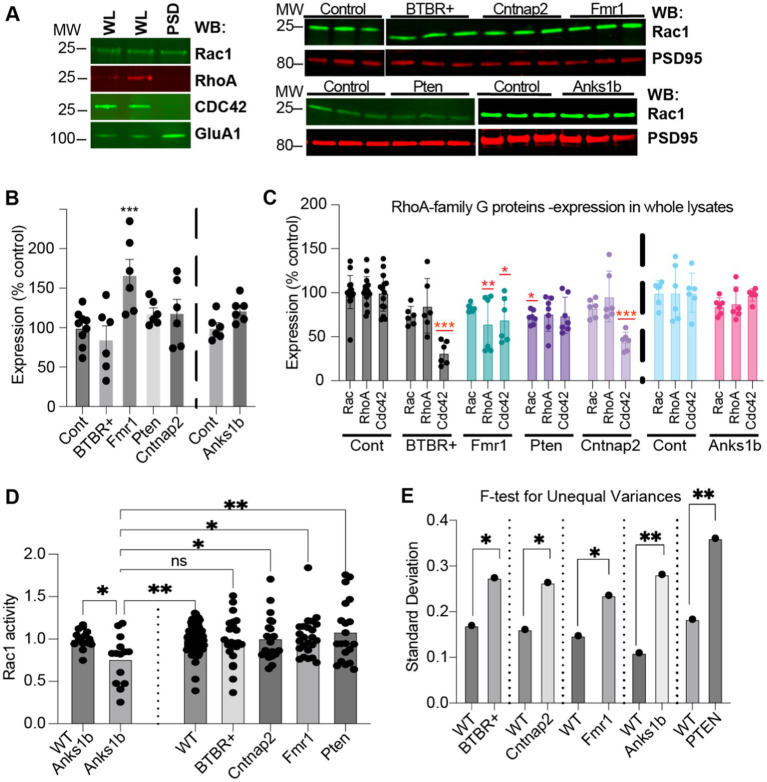
Validation of RhoA family GTPases dysfunctions in ASD mouse models **(A)** (Left) Rac1 was the only Rho GTPase detected in whole lysate (WL) and synaptic (PSD enriched) fractions (5 μg each). (Right) Western blots (5 μg PSD enriched fraction) showing Rac1 and synaptic marker PSD95 in the different models. **(B)** Quantitation shows increased Rac1 in synaptic fractions from *Fmr1* KO, but not from other mouse models, compared to controls. **(C)** Quantitation of RhoA family GTPases in total lysates shows differences throughout. Expression of Cdc42 in total lysate was reduced for all models except in *Anks1b* Het, where no significant change was observed (30 μg whole lysate). **(D)** G-LISA showing difference in fold-change in active Rac1 compared to appropriate controls. Only *Anks1b* showed significant reduction in active Rac1. **(E)** *F*-test calculations for unequal variances show significance. Bar graphs show mean ± SEM, *n* = 3–10 samples for each mouse model and wild-type control strain, Student’s *t*-test, *p < 0.05 **p < 0.01 ***p < 0.001. For **B,C** sample values are compared to controls.

To validate our proteomic and bioinformatic analyses, we performed biochemical analyses for one altered cellular pathway among all identified. Across all mouse models tested, RhoA family G-protein signaling pathways are involved in all top canonical pathway hits (Figure 3A) including Synaptogenesis Signaling Pathway, Signaling by Rho family GTPases, Rac Signaling, RhoGDI signaling, and Regulation of Actin-based motility by Rho. Bioinformatic predictions validate previous studies as Rho GTPases have crucial roles in synaptic function and have been investigated extensively in both human-based experiments and mouse models of ASD (Guo et al., 2020). Directionality of changes was largely consistent among mouse models, predicting activated signaling by Rho GTPases and inhibited regulation by RhoGDI in *Pten*, *Cntnap2*, BTBR+, and *Fmr1* mice, but with opposite changes observed in the *Anks1b* mice (Figure 3A). Depictions of the Rac1 signaling pathways altered in the ASD models show that receptor tyrosine kinases and other synaptic pathways primarily drive changes in Rac activity (Supplementary Figure 6). Components of the WAVE complex and other regulators of actin nucleation and polymerization feature prominently among altered molecules in this pathway. Western blots for Rho family G-proteins Rac1, RhoA, and Cdc42 in synaptic fractions detected Rac1, but not RhoA or Cdc42 in synaptic fractions (Figure 4A). This corroborates the MS analyses showing that Rac1 was identified with more unique peptides in enriched PSD fractions compared to RhoA and CDC42 (Supplementary Table 1). Rac1 expression was increased in the *Fmr1* KO model in synaptic fractions, but largely unchanged across all models (Figures 4A,B). Western blots of whole brain lysates reveal significant changes in several Rho family GTPases across models (Figure 4C). For a more informative test measuring function, we performed G-protein activation ELISAs (G-LISAs) to directly measure Rac1 activity in total hippocampal lysates. As predicted by bioinformatic analyses, Rac1 activity in *Anks1b* model mice was substantially downregulated compared to controls, as well as significantly reduced compared to *Cntnap2*, *Pten*, and *Fmr1* models (Figure 4D). While Rac1 activity in *Pten, Fmr1, Cntnap2*, and BTBR+ mice was not significantly upregulated as predicted, a difference in the variance of the WT and ASD dataset was striking. We used F-test calculations for unequal variances to show the differences were statistically significant (Figure 4E).

## Discussion

ASDs are among the most heritable neuropsychiatric conditions, with early epidemiological studies estimating concordance rates of ~90% in monozygotic twins, compared to ~10% for dizygotic twins (Steffenburg et al., 1989; Bailey et al., 1995; Hallmayer et al., 2011). Hundreds of genes and chromosomal loci for ASD susceptibility have been reported, suggesting a highly heterogeneous genetic architecture (Chen et al., 2015; de la Torre-Ubieta et al., 2016). While these studies highlight the notion that there is no single “autism gene,” functional and bioinformatic analyses of genetic studies have identified convergent cellular pathways, including those regulating transcription (Sanders et al., 2012; de Rubeis et al., 2013), excitatory/inhibitory (E/I) balance (Blatt et al., 2001; Hussman, 2001; Rubenstein and Merzenich, 2003; Gao and Penzes, 2015; Nelson and Valakh, 2015), and especially synaptic function (Bucan et al., 2009; Glessner et al., 2009; Hussman et al., 2011; Buxbaum et al., 2012; Sanders et al., 2012; Yu et al., 2013; Brett et al., 2014; Buxbaum et al., 2014; Cukier et al., 2014; Iossifov et al., 2014; Pinto et al., 2014; Ronemus et al., 2014; Toma et al., 2014; Yuen et al., 2015). Autism gene databases show clear enrichment for synaptic pathways, and syndromic ASD mouse models are primarily associated with mutations in synaptic proteins or their regulators, such as SHANKs, neurexins, neuroligins, SynGAP1, FMRP, and TSC1/2 (Pinto et al., 2014; Yoo, 2015; Ramaswami and Geschwind, 2018). Indeed, most (if not all) identified ASD mouse models such as those for Fragile X (Bhakar et al., 2012), tuberous sclerosis (Auerbach et al., 2011), Angelman (Mabb et al., 2011) Phelan McDermid (Sarowar and Grabrucker, 2016), and *ANKS1B* syndromes show deficits in diverse forms of synaptic function as well as altered neuronal and synaptic morphology (Tindi et al., 2015; Carbonell et al., 2019). Here, we compared the hippocampal postsynaptic proteomes from five mouse models for autism that display synaptic dysfunction, including altered synapse formation, excitatory/inhibitory balance, synaptic plasticity, and glutamatergic signaling (Hulbert and Jiang, 2016; Varghese et al., 2017; Bagni and Zukin, 2019; Carbonell et al., 2019). The goal of this work was to test the hypothesis that common structural and functional deficits among diverse ASD mouse models are caused by similar underlying synaptic deficits and ASD-linked susceptibility factors that ultimately converge on common signaling pathways. By leveraging widely studied ASD mouse models, we hope to identify high-value therapeutic targets for autism.

Wide-spread genetic- and transcriptomic-based screening and comparative studies of disease models suffer from important caveats that limit their interpretability. Often, little is known about how disease-linked genes or other chromosomal loci ultimately correlate to protein abundance and function. Moreover, multiple studies confirm the weak to nil correlation between transcript and protein abundance (Greenbaum et al., 2003; Maier et al., 2009). Transcriptomes display 100-fold ranges in translation efficiency (Ingolia et al., 2009), proteomes reveal >1,000-fold ranges in half-lives (Doherty et al., 2009), and coupled transcriptomic and proteomic analyses reveal that proteins are ~900 times more abundant than corresponding mRNAs, but with ratios that span over five orders of magnitude (Schwanhäusser et al., 2011). Finally, transcriptomic methods are unlikely to provide meaningful information for ASDs such as Angelman syndrome, Fragile X syndrome, and tuberous sclerosis, where the primary contributors to disease etiology are thought to be altered regulation of protein translation and degradation. These important caveats are often ignored in discovery-based genomic research (Wang et al., 2017; Hoogendijk et al., 2019). To overcome these concerns, we have taken a quantitative proteomic approach based on 10-plex Tandem Mass Tags (TMT; Erdjument-Bromage et al., 2018). By directly measuring synaptic protein abundance, we bypass the concerns about poor transcript and protein correlation, as well as the need to understand the complex functional consequences of altered gene expression.

We have used IPA (Qiagen) to analyze the proteomic data and generate inferences on pathways altered in each disease model. IPA derives functional insights and pathway nomenclatures from a continuously updated knowledge base drawn from the literature (Krämer et al., 2014). While this implies a broad context for functional annotations, it also means that proteins are likely to be associated with scientific trends. Perhaps not surprisingly given the current pandemic, several Diseases and Functions associated with the ASD models are related to viral infections (Supplementary Figure 3). Moreover, there is the possibility for missed hits given the lack of comprehensive annotations for cellular and molecular pathways. One example is the surprising finding that the most downregulated proteins across four of the five ASD models were associated with oligodendrocyte function (Supplementary Table 2). The appearance of proteins related to myelin was surprising in our synaptic fractions but may reflect the presence of neuron-oligodendrocyte synapses that may co-purify using our methods. Despite these limitations, IPA lists the specific proteins and their fold-changes leading to the assigned pathways. For example, the prediction for activated Oxidative phosphorylation across ASD models is primarily driven by increases in the abundance of ATPases and different subunits of the cytochrome c oxidases and the NADH:ubiquinone oxidoreductase. Changes in synaptogenesis signaling pathways were driven by both increases and decreases in the abundance of regulators of the actin cytoskeleton which include several small G-proteins (Supplementary Table 4).

Regulatory networks predicted by synaptic changes in each model reveal both novel results and patterns consistent with the literature (Figures 3, 4; Supplementary Figures 1-4; Supplementary Tables 1-4). In the *Fmr1* KO model, fold-changes reveal that most altered proteins were upregulated as seen by the rightward skew of the ratio distributions (Figure 2A), which is consistent with the primary role of FMRP as a translational repressor (Darnell et al., 2011). In the model of constitutive *Pten* haploinsufficiency, synaptic dysfunction is likely due to neuronal *Pten*, since altered synaptic composition was also observed in a neuron-specific conditional knockout mouse that displayed changes in activity patterns and repetitive behavior (Lugo et al., 2014). Importantly, we directly compared synaptic proteomes in parallel, a necessary approach for finding shared phenotypes and broadly applicable therapeutic targets (Sestan and State, 2018; Silverman and Ellegood, 2018). Drawn from internally controlled measurements, our results demonstrate quantifiable differences in synaptic composition that support the presence of molecular subtypes in ASD rather than universal changes in the same direction.

In the five autism mouse models chosen for this study, changes in the synaptic proteome highlighted Rho family small GTPase signaling as a commonly altered cellular pathway (Figures 3, 4). These results complement a growing body of evidence showing that altered activation of Rho GTPases is an important mechanism of disease in autism and other neurodevelopmental disorders (Pinto et al., 2010; Zeidán-Chuliá et al., 2013; Guo et al., 2020). Functional validation results showed significantly reduced active Rac1 in *Anks1b* mice compared to wildtype, and compared to *Pten*, *Fmr1,* and *Cntnap2* mice, which was consistent with proteomic analysis (Figures 3, 4). These results suggest that downregulation of Rac1 pathways may inhibit the processes that require GTP-bound Rac1, thereby potentially causing some of the manifestations of *ANKS1B* syndrome, including ASD. Consistent with our findings that synaptic proteins in the Rac pathway (Supplementary Figure 6) and Rac1 itself is differentially regulated in ASD mouse models (Figure 4), further evidence continues to emerge that Rac1 signaling plays an important role in the pathobiology of ASD and other neurodevelopmental disorders. Rac1 mutations associated with intellectual disability impair synaptic plasticity (Tian et al., 2018), and variants in the RhoGEF *TRIO* can cause ASD, intellectual disability, schizophrenia, or macrocephaly, with severe phenotypes predicted by Rac1 overactivation (Katrancha et al., 2017; Sadybekov et al., 2017; Barbosa et al., 2020). Both up- and down-regulation of Rac1 in mice causes social deficits, an important symptom of ASD (Ma et al., 2022). Downstream of Rac1 in actin polymerization, mutations in *PAK*s and *WASF1* can cause macrocephaly, seizures, intellectual disability, or ASD (Ito et al., 2018; Horn et al., 2019). Overall, Rac1 pathways are commonly dysregulated in ASD and other neurodevelopmental disorders and are therefore attractive targets for pharmacotherapy (Zamboni et al., 2018; Guo et al., 2020).

Caveats to our work include comparisons at a single developmental timepoint and single brain region. Models of ASD show behavioral and biological phenotypes that differ based on the spatiotemporal targeting of the gene (Del Pino et al., 2018). A proteomic study of cortical synapses noted smaller differences in *Fmr1* KO mice after 3 weeks of age (Tang et al., 2015), and both Shank3 and Syngap1 show changes in interaction partners throughout development (Li et al., 2016, 2017). Moreover, we focused on hippocampal synapses, but there are important roles for the neocortex, striatum, and cerebellum in the pathophysiology of ASD animal models (Golden et al., 2018). Fractionation of the PSD limits our findings to synaptic profiles in glutamatergic neurons, but ASD risk genes also influence interneuron development (*Cntnap2*; Peñagarikano et al., 2011) and oligodendrocyte maturation (*Pten*; Lee et al., 2019). The number of isobaric tags available in the TMT set limited the number of simultaneous comparisons possible by mass spectrometry. We therefore had to analyze the biological replicates as three independent experiments, which increases variability. To minimize this variability, fold-ratios were calculated only between samples in the same MS run. Future experiments using increased isobaric tags such as the new TMT16-plex system could be used to compare proteomes across cell types and developmental stages, and additional ASD models (Stessman et al., 2016; Sestan and State, 2018; Iakoucheva et al., 2019).

Understanding the molecular mechanisms underlying ASDs is critical for the development of novel therapies. Our proteomic approach overcomes critical confounds associated with gene-based studies of ASD etiology that improperly equate transcript levels, epigenetic modifications, single-nucleotide polymorphisms (SNPs), or copy number variations (CNVs) to changes in protein abundance. This work shows that synaptic proteomes, as identifiable and quantifiable phenotypes of diverse ASD models, can lead to the identification of molecular convergence. We propose that this can lead to important insights into ASD etiology and yield high-value targets to pursue for future therapies.

## Data availability statement

The raw mass spectrometry data generated during this study are available at MassIVE (UCSD), https://massive.ucsd.edu/ProteoSAFe/static/massive.jsp under the deposition number MSV000091848.

## Author contributions

AC, CF-C, ID, and BJ designed and performed postsynaptic fractionation in autism mouse models. HE-B and TN performed tandem-mass-tag mass spectrometry and protein identification. AC and ID performed bioinformatics analysis and Western blots. ID and SD performed the GLISA assays. AC-A and DP developed and characterized Pten haploinsufficiency mouse model. AC, CF-C, ID, SD, and BJ interpreted results and wrote the paper. All authors contributed to the article and approved the submitted version.

## Funding

This work was supported by NIH R01AG039521, NIH R01NS118820, and NIH R56MH115201 to BJ, T32GM007288 to AC, NIH S10RR027990 to TN, and NIH R01MH108519 to DP. Significant support for this work came from the Rose F. Kennedy Intellectual and Developmental Disabilities Research Center (IDDRC), which is funded through the center grant NIH U54HD090260.

## Conflict of interest

The authors declare that the research was conducted in the absence of any commercial or financial relationships that could be construed as a potential conflict of interest.

## Publisher’s note

All claims expressed in this article are solely those of the authors and do not necessarily represent those of their affiliated organizations, or those of the publisher, the editors and the reviewers. Any product that may be evaluated in this article, or claim that may be made by its manufacturer, is not guaranteed or endorsed by the publisher.

## Supplementary material

The Supplementary material for this article can be found online at: https://www.frontiersin.org/articles/10.3389/fnagi.2023.1152562/full#supplementary-material
