# Supplementary material for: Comparing synaptic proteomes across five mouse models for autism reveals converging molecular similarities including deficits in oxidative phosphorylation and Rho GTPase signaling
Source: Front Aging Neurosci. 2023 May 15;15:1152562. doi: 10.3389/fnagi.2023.1152562 (PMC10225639; doi:10.3389/fnagi.2023.1152562)

Supplementary Table 1. Raw intensity data, gene names, peptides identified, and Q-values (p-values adjusted for multiple comparisons) and other information for all proteins identified in experiments 1 (sheet 1), 2 (sheet 2), and 3 (sheet 3) from the TMT-MS analyses. The last sheet contains the total number of proteins identified in each experiment and their overlap with proteins in the SynGO and G2Cdb synaptic databases.

Supplementary Table 2. Fold ratios calculated for all proteins identified in experiments 1, 2, and 3 and one-sample T-test calculations for all protein ratios calculated. These proteins represent the filtered dataset that was subsequently analyzed IPA, containing proteins that were identified and quantified with at least 3 unique peptides, Q-values of 0, and that were identified and quantified in at least two replicates. In the second sheet, all proteins were sorted by the sum of the ratios in all ASD models.

Supplementary Table 3. Top Canonical pathways, Upstream regulators, and Diseases and Functions identified for each ASD model.

Supplementary Table 4. Proteins and fold-changes for each ASD model that comprise the identified cellular and molecular pathways in canonical pathways, upstream regulators, and diseases and functions.

Supplementary Fig 1. Canonical pathways for each individual ASD model showing the 20 most upregulates and downregulated pathways.

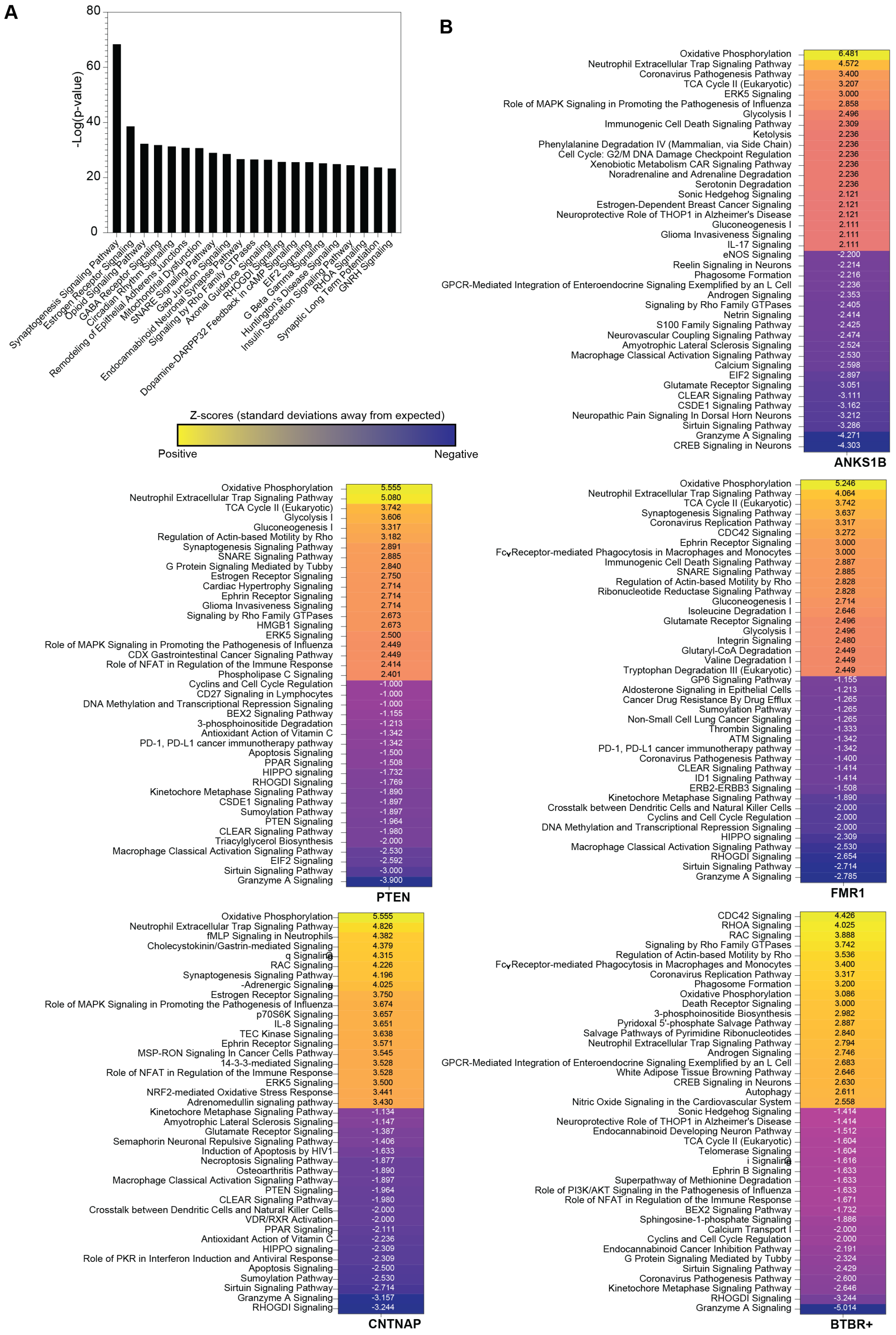

Supplementary Fig 2. Upstream regulators pathways for each individual ASD model showing the 10 most upregulates and downregulated pathways

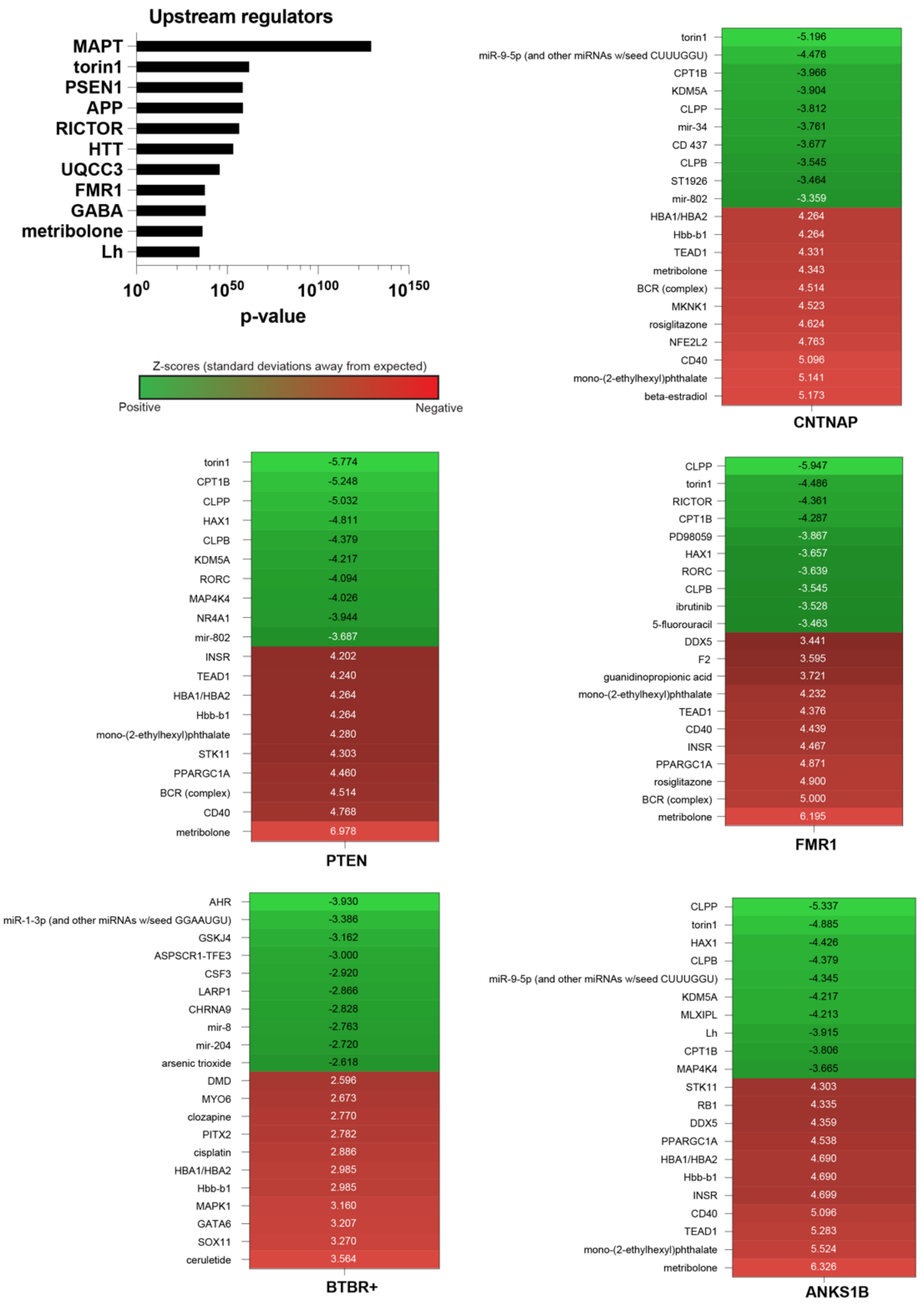

Supplementary Fig 3. Most significant diseases and functions identified for each ASD model.

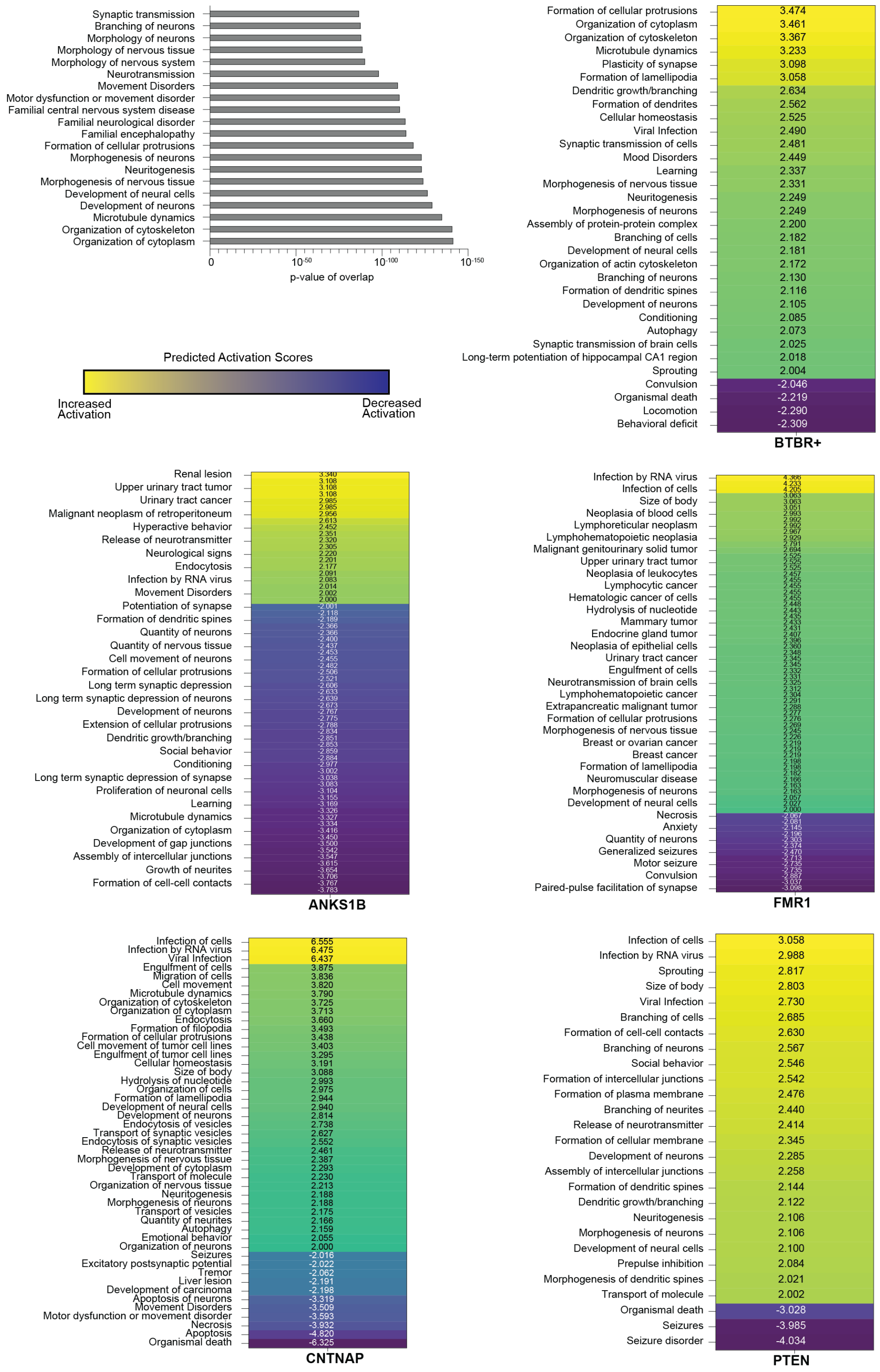

Supplementary Fig 4. Upstream regulators and Diseases and Functions for Comparison analyses for all ASD models.

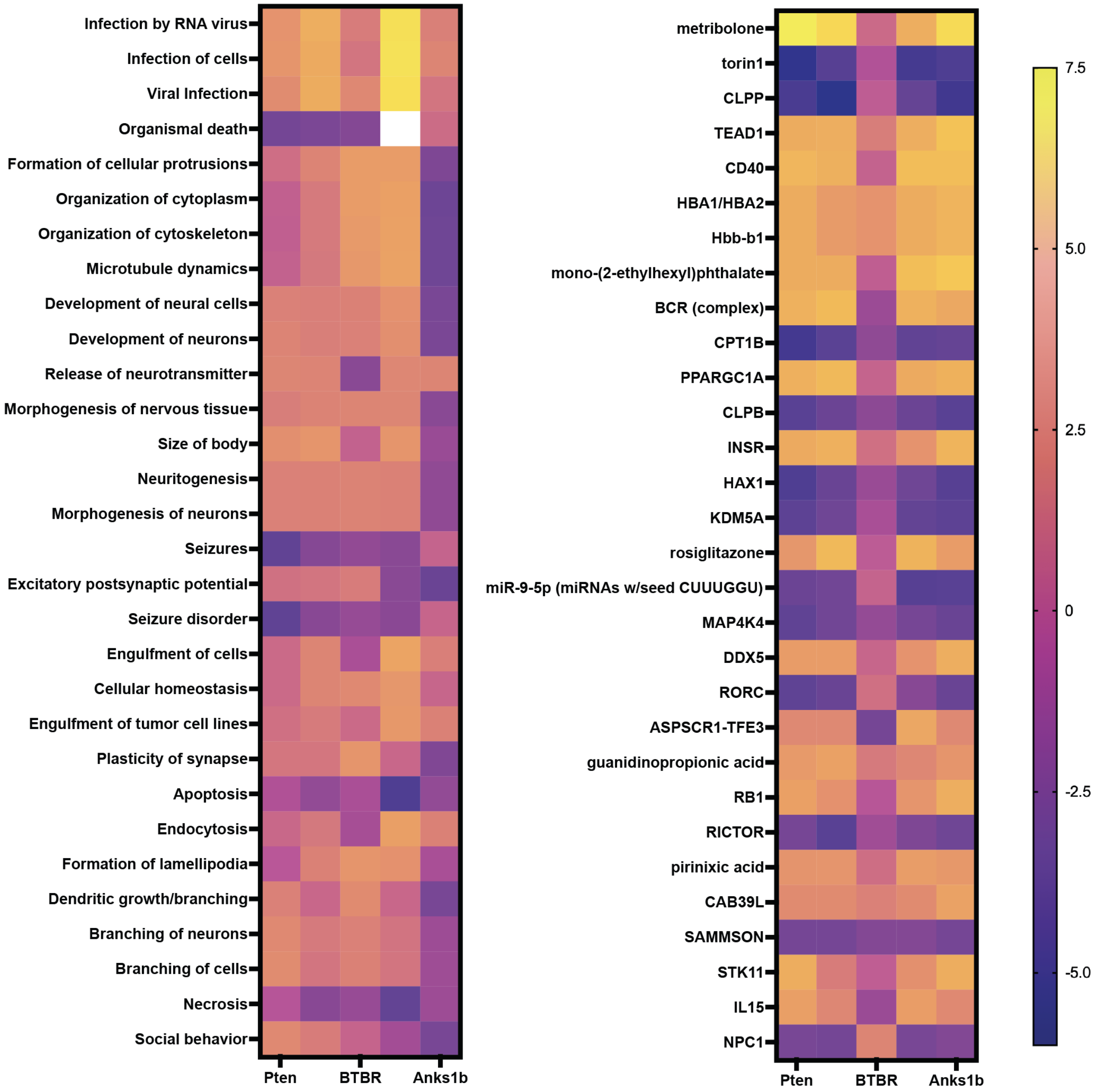

Supplementary Fig 5. TMT labeling efficiency.
A) Workflow of proteomics experiments from sample preparation to peptide identification. **B,D,F**) Distribution of protein intensities for the 10 reporter ions in experiments 1, 2, and 3, each showing a normal distribution. **C,E,G**) Scatterplots comparing reporter ion intensities for proteins between each of the 10 TMT channels. Spearman's rank-order correlation, $\rho > 0.96$ for all TMT channel comparisons in all experiments.

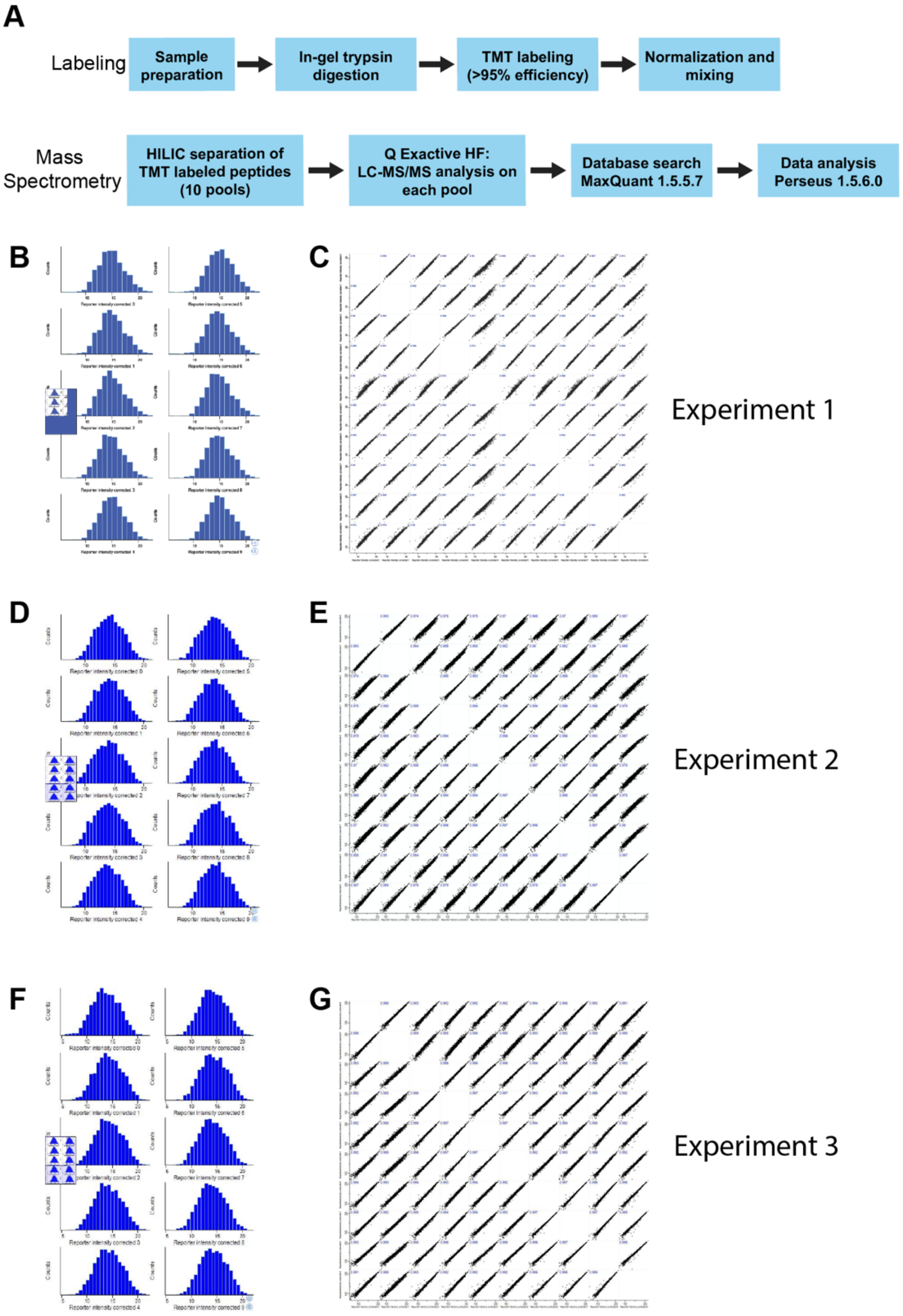

Supplementary Figure 6. Rac Signaling Pathways generated in IPA during Comparison Analyses are differentially regulated across mouse models of autism. Protein components of the Rac Signaling Pathway in IPA are up- or downregulated in the postsynaptic proteomes of ASD mouse models, leading to predicted effects on pathway activation in Pten, Cntnap2, BTBR+, Fmr1, and Anks1b Het mice.

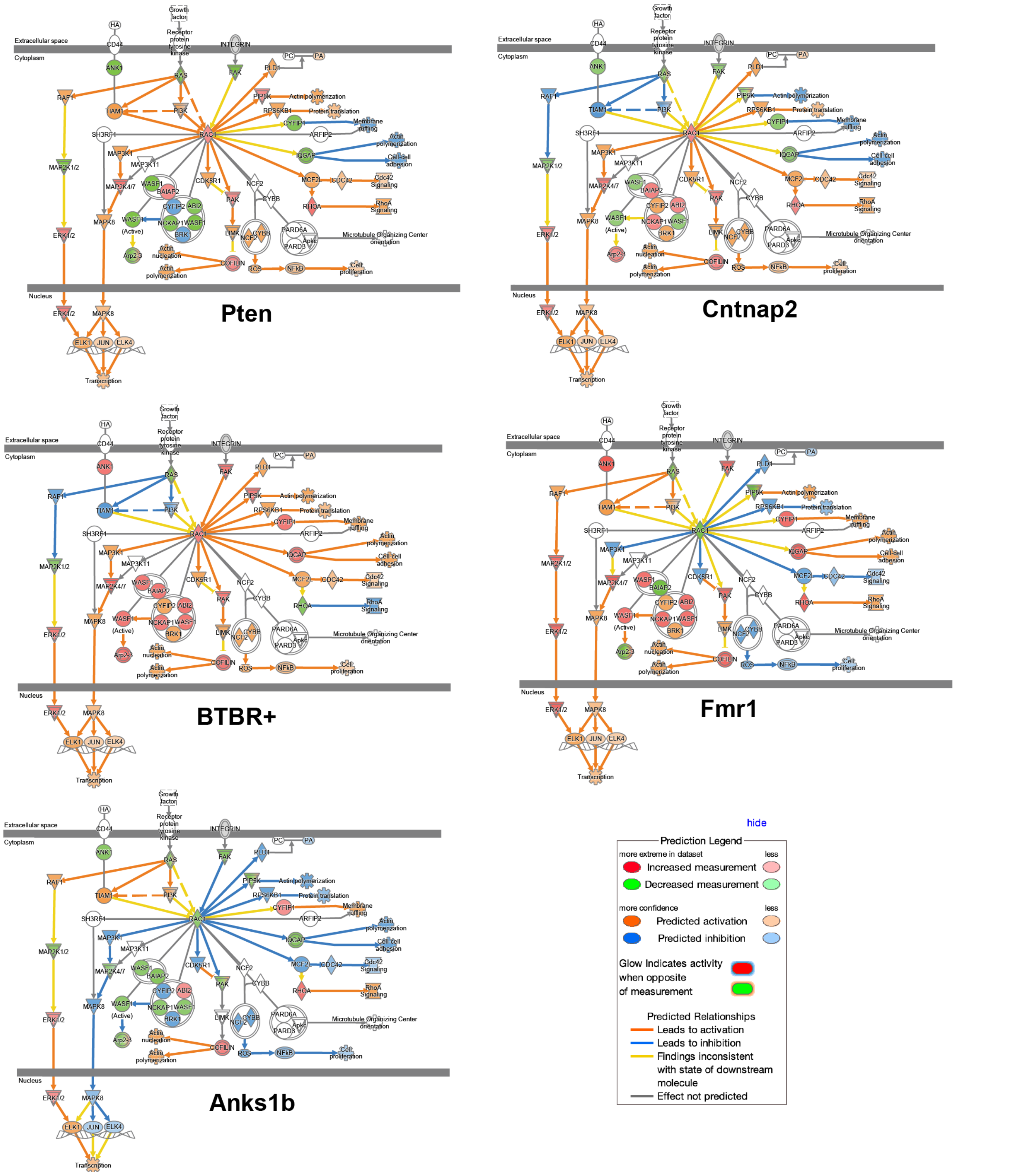

Supplement: Supplementary file 1 [file Data_Sheet_1.PDF]
